# A global clinicopathologic and molecular portrait of spindle cell/sclerosing rhabdomyosarcoma with emphasis on retroperitoneal cases

**DOI:** 10.3389/fonc.2026.1735547

**Published:** 2026-04-01

**Authors:** Xiaoying Zhang, Haining Zheng, Ming Zhang

**Affiliations:** 1 Department of Pathology, Peking University International Hospital, Beijing, China; 2 Department of Ultrasound, Peking University International Hospital, Beijing, China

**Keywords:** individual patient data, meta-analysis, retroperitoneum, rhabdomyosarcoma, sclerosing, spindle cell

## Abstract

**Introduction:**

Spindle cell and sclerosing rhabdomyosarcoma (Sc/SRMS) are rare histologic subtypes of rhabdomyosarcoma, increasingly recognized for their distinct molecular profiles and aggressive clinical behavior. Retroperitoneal involvement is exceptionally uncommon and poorly characterized. To characterize the clinical, pathological, and molecular features of retroperitoneal Sc/SRMS through a combined institutional case series and individual patient data (IPD) meta-analysis.

**Methods:**

We retrospectively analyzed 12 adult patients with retroperitoneal Sc/SRMS treated at Peking University International Hospital between 2014 and 2023. Clinical data, imaging, histopathology, immunohistochemistry, and MYOD1 mutation status were evaluated. In parallel, we conducted a systematic review and IPD meta-analysis of 136 publications, yielding 403 unique Sc/SRMS cases. Data were extracted on demographics, tumor characteristics, treatment, molecular alterations, and outcomes.

**Results:**

All institutional cases presented with large, high-grade retroperitoneal tumors, frequently invading adjacent organs. MYOD1 mutations were detected in 33.3% of patients, and MyoD1 immunopositivity exceeded myogenin (92% vs. 42%, P<0.05). All patients experienced local recurrence; 83.3% died of disease. Kaplan-Meier analysis revealed a 2-year survival rate of 40%. The IPD meta-analysis identified six additional retroperitoneal cases, none of which reported molecular data. Across the full dataset, MYOD1 and PIK3CA mutations were observed in 95.5% and 100% of tested cases, respectively. Fusion genes such as FUS::TFCP2 and EWSR1::TFCP2 were also prevalent. Overall, 34.7% of patients died of disease and 41.7% developed local recurrence.

**Discussion:**

Retroperitoneal Sc/SRMS exhibits a highly aggressive clinical course, marked by extensive local invasion, high recurrence rates, and poor survival. Our institutional series represents the largest molecularly characterized cohort of retroperitoneal cases to date. Integration with IPD meta-analysis supports the importance of MYOD1 mutation and highlights the frequency of PIK3CA co-mutations. However, these frequency estimates are derived from a subset of tested cases and should be interpreted within the context of variable molecular data reporting across studies. These findings highlight the potential value molecularly guided treatment strategies in this rare and lethal disease subtype.

## 1 Introduction

Rhabdomyosarcoma (RMS) is the most common soft tissue sarcoma of childhood and adolescence, but it also occurs, albeit rarely, in adults (1, 2). Among its histologic subtypes, spindle cell and sclerosing RMS (Sc/SRMS) represent recently delineated variants with distinctive clinical, morphological, and molecular features (1, 2). Initially regarded as rare morphological patterns within embryonal RMS, both spindle cell and sclerosing types are now recognized by the World Health Organization as distinct subtypes (3), particularly due to their association with specific genetic alterations and divergent clinical behaviors across age groups.

Sc/SRMS poses unique diagnostic and therapeutic challenges (4–6). Histologically, these tumors can mimic a wide range of benign and malignant spindle cell neoplasms, often leading to misdiagnosis, particularly in adult patients and in uncommon anatomical locations (7–9). Recent studies have shown that Sc/SRMS frequently harbor recurrent MYOD1 mutations—most notably the canonical L122R variant—often accompanied by PIK3CA co-mutations (10–13). These alterations are associated with aggressive behavior and poor outcomes, particularly in adult and extra-cranial presentations. Moreover, gene fusions such as FUS::TFCP2 (14–18) and EWSR1::TFCP2 (6, 19, 20) have emerged as molecular hallmarks in a subset of cases, particularly those arising in the head and neck region. Despite these advances, retroperitoneal Sc/SRMS remains exceptionally rare, with only isolated cases reported in the literature (21–26).

To date, no study has comprehensively characterized the clinical, pathological, and molecular landscape of retroperitoneal Sc/SRMS (22–24). Furthermore, large-scale, patient-level syntheses of the broader Sc/SRMS population—across age groups and anatomical sites—are lacking. Addressing this gap is critical, given the poor prognosis and therapeutic ambiguity associated with these tumors.

In this study, we present a dual-pronged investigation comprising (1) a single-institution retrospective case series of 12 adult patients with retroperitoneal Sc/SRMS, and (2) an individual patient data (IPD) meta-analysis of 403 cases extracted from 136 published reports. By integrating granular clinical, radiological, immunohistochemical, and molecular data, we aim to (a) delineate the unique features of retroperitoneal Sc/SRMS, (b) situate these findings within the broader spectrum of Sc/SRMS, and (c) provide a high-resolution view of prognostic factors that may inform future clinical management and research directions.

## 2 Materials and methods

### 2.1 Institutional case series

#### 2.1.1 Design & clinical data

This study included a retrospective case series of 12 patients diagnosed with spindle cell or sclerosing rhabdomyosarcoma (ScRMS/SRMS) of the retroperitoneum at Peking University International Hospital. Data were retrieved from the hospital’s electronic medical records system, covering the period from December 24, 2014, to October 31, 2023. Data extraction was conducted on October 31, 2023. Only de-identified datasets were accessed, with patient IDs replacing identifiable information. No member of the research team had access to personally identifiable information during or after the study. Ethical approval was obtained from the Ethics Committee of Peking University International Hospital (No. 2018–062 [BMR]), which waived the requirement for informed consent because the study was retrospective and relied solely on fully anonymized data. All procedural aspects of this investigation were conducted in accordance with the 2013 revision of the Declaration of Helsinki.

Among the 12 cases, eight patients underwent their initial surgery at our institution, while four presented with recurrence following surgery at outside facilities. Clinical information was collected from hospital records and follow-up documentation, including patient age, gender, tumor location and laterality, medical and treatment history, recurrence status, metastasis, and molecular testing results. The diagnosis of ScRMS/SRMS was based on histopathological morphology, immunohistochemical staining, and genetic testing. All cases were independently verified by two senior pathologists in accordance with the diagnostic criteria of the World Health Organization.

#### 2.1.2 Immunohistochemical methods

Tumor tissue specimens were fixed in 10% neutral formalin, dehydrated, embedded in paraffin, and sectioned at a thickness of 4 µm. Hematoxylin and eosin (HE) staining was performed, followed by immunohistochemical staining using the EnVision two-step method. Positive and negative controls were established. The antibodies used included Desmin, Myogenin, MyoD1, SMA, CD34, S-100, MDM2, CDK4, P16, H3K27Me3, and P53. All antibodies and the EnVision kit were sourced from Beijing Zhongshan Jinqiao Technology Co., Ltd. The standard protocol included dewaxing, high-pressure antigen retrieval, inactivation of endogenous enzymes, sequential application of primary antibodies, enhancer, enzyme-labeled anti-mouse/rabbit polymer, DAB chromogen, hematoxylin counterstain, dehydration, and mounting.

Staining localization and scoring followed standardized criteria: myogenin, MyoD1, MDM2, CDK4, and H3K27Me3 were assessed in the nucleus; Desmin and SMA in the cytoplasm; CD34 in both cytoplasm and membrane; and S-100, p16, and P53 in both nucleus and cytoplasm. P53 staining was interpreted as wild-type (heterogeneous moderate nuclear staining), overexpressed (diffuse strong nuclear staining), or null (complete absence of staining). Other markers were evaluated using the Fromowitz scoring system: percentage of positive tumor cells was scored from 0 to 3 (based on <25%, 26–50%, 51–75%, >75%), and staining intensity from 0 to 3 (no color to strong brown). The final IHC score (0–6) was categorized as negative (0), + (1–2), ++ (3–4), and +++ (5–6). Two senior attending physicians independently reviewed each slide in a double-blind manner.

#### 2.1.3 Molecular testing for MYOD1

Molecular analysis of MYOD1 mutations was performed via Sanger sequencing. The transcript reference was NM_002478.5 (MANE). PCR targeted the known hotspot in exon 1 using specific primer sets: MYOD1 E1-1-F, MYOD1 E1-1-R, MYOD1 E1-2-F, MYOD1 E1-2-R, MYOD1 E1-3-F, and MYOD1 E1-3-R. All identified mutations were somatic. The workflow included bacterial precipitation and resuspension, cell lysis and protein precipitation, DNA extraction and purification using phenol/chloroform, ethanol precipitation, and resuspension in TE buffer. DNA was denatured, and sequencing reactions were set up with oligonucleotide primers, DTT, dNTPs, and modified T7 DNA polymerase. Termination reactions were performed using ddNTPs, followed by electrophoresis on gradient polyacrylamide gels. Gels were dried and autoradiographed for sequence interpretation.

#### 2.1.4 Statistical analysis

All statistical analyses for the institutional case series were conducted using SPSS version 26.0. Descriptive statistics were calculated for all clinical variables. Survival outcomes were analyzed using the Kaplan-Meier method. Fisher’s exact test was used to compare categorical variables between subgroups. A two-sided p-value <0.05 was considered statistically significant.

### 2.2 Systematic review and individual patient data meta-analysis

#### 2.2.1 Search strategy and eligibility criteria

A systematic review of the literature was performed to identify case reports and case series describing spindle cell and/or sclerosing rhabdomyosarcoma, from database inception to June 20, 2025. Searches were conducted in PubMed, Embase, and Web of Science using the following search terms (adjusted per database): “spindle cell rhabdomyosarcoma” OR “spindle-cell rhabdomyosarcoma” OR “sclerosing rhabdomyosarcoma”. Studies were eligible if they reported individual patient-level data (27). Reports limited to aggregate data without extractable individual information were excluded. Duplicate publications, review articles, and studies without histologically confirmed ScRMS/SRMS were also excluded. Study selection was performed independently by two reviewers (XZ, HZ) through two phases: title/abstract screening followed by full-text review against eligibility criteria. Disagreements were resolved through discussion or consultation with a third reviewer (MZ). The protocol was registered on PROSPERO (ID: 1088186).

#### 2.2.2 Data extraction and variables

From each eligible publication, patient-level data were extracted into a standardized database. Extracted variables included country, year of publication, age, gender, rhabdomyosarcoma subtype (spindle, sclerosing, or mixed), laterality, tumor location, three-dimensional size (in cm), primary therapy, clinical outcome (NED, DOD, AWD, etc.), follow-up duration (in months), T2-weighted and T1-weighted MRI signal intensity, contrast-enhancement pattern on CT or MRI, Ki67 index, immunohistochemistry findings, fusion status (e.g., VGLL2-CITED2, TEAD1-NCOA2), FISH results, and genetic mutations (e.g., MYOD1, PIK3CA). When three-dimensional size was reported, tumor volume was calculated using the largest given value (Volume, 0.523 × L × W × H). If a study presented multiple cases but only provided summary statistics, those cases were excluded. Where specific variables of interest (e.g., tumor size, follow-up duration, IHC or molecular results) were not reported, these data points were coded as ‘not available’ and excluded from the respective descriptive analyses. Additionally, no attempts were made to impute missing data or contact study authors, given the inherent nature and large volume of case report literature.

#### 2.2.3 Data analysis

Descriptive analyses were conducted for all extracted patient-level variables. Due to the nature of the source material (case reports/series) and the heterogeneous reporting, inferential statistical tests were not applied to the pooled data from the systematic review. However, pooled descriptive statistics were used to characterize the overall landscape of ScRMS/SRMS presentation, treatment, and outcome. Data from the institutional case series were analyzed in parallel and reported alongside the meta-analytic findings to enhance contextual interpretation. Given the inherent clinical and methodological heterogeneity of case reports and small case series, formal quantitative synthesis (including I² heterogeneity testing and meta-regression) was not performed. Subgroup analyses were therefore limited to descriptive comparisons of aggregated data.

## 3 Results

### 3.1 Institutional case series of retroperitoneal Sc/SRMS

**Figure 1:**
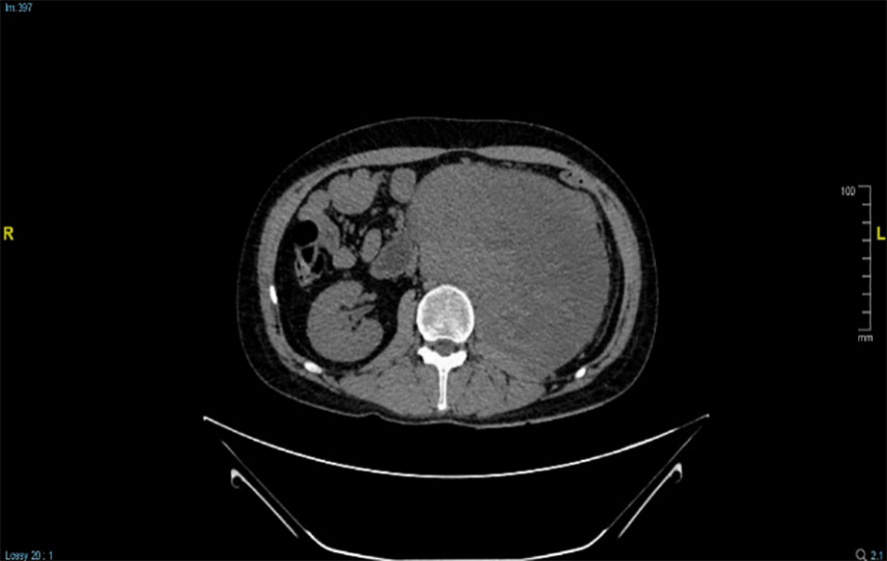
Enhanced CT showing retroperitoneal rhabdomyosarcoma.

**Figure 2:**
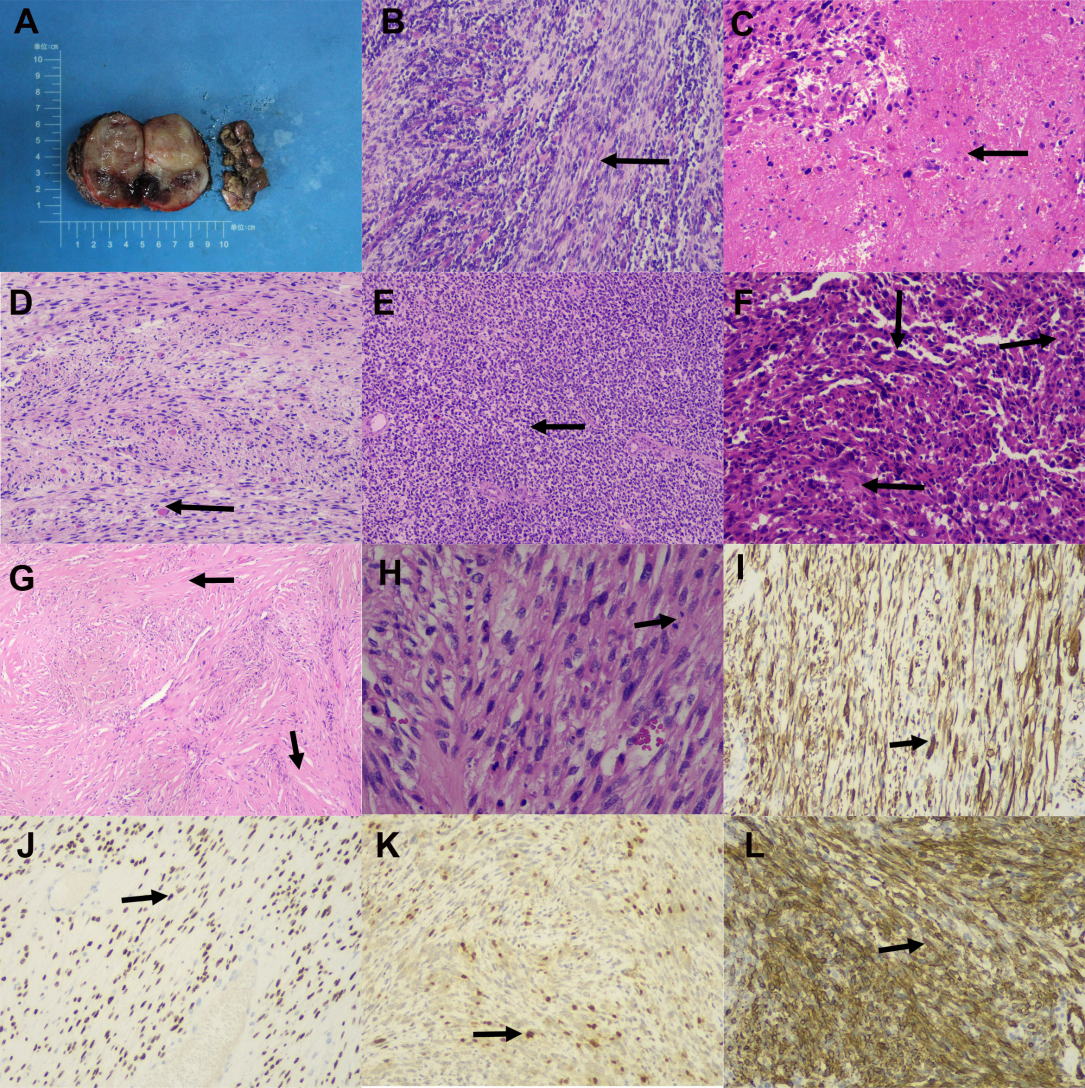
Gross and microscopic manifestations of retroperitoneal spindle cell/sclerotic rhabdomyosarcoma. **(A)** Gross appearance of retroperitoneal spindle cell/sclerosing rhabdomyosarcoma. **(B)** Tumor cells arranged in fascicular bundles (arrow) (HE, 100×). **(C)** Foci of extensive sheet-like necrosis (arrow) observed in some cases (HE, 100×). **(D)** Striated myoblasts (arrow) identified in most tumors (HE, 100×). **(E)** .Presence of embryonal components (arrow) in some cases (HE, 100×). **(F)** Some tumors showing polymorphic components with markedly atypical cells (arrows) (HE, 100×). **(G)** Sclerosing subtype characterized by prominent hyalinization and stromal sclerosis (arrows) (HE, 40×). **(H)** Frequent mitotic figures (arrow) (HE, 200×). Immunohistochemical staining demonstrated positivity for **(I)** Desmin with cytoplasmic staining (arrow), **(J)** MyoD1 with nuclear staining (arrow), **(K)** Myogenin with nuclear staining (arrow), and **(L)** .SMA with cytoplasmic staining (arrow) (EnVision, 100×).

**Figure 3:**
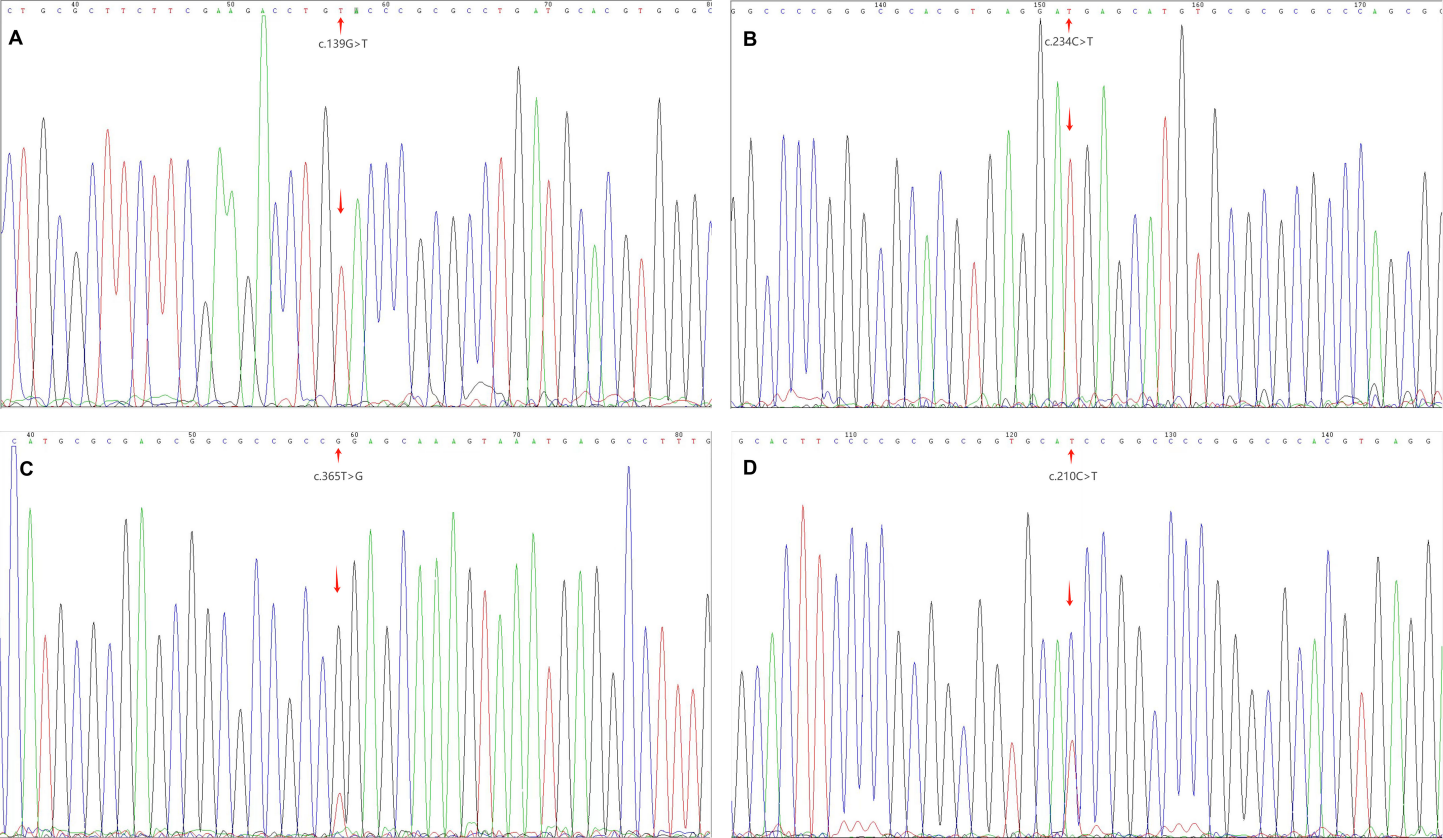
MyoD1 gene mutation **(A, B)** show homozygous mutations (arrow); **(C, D)** show heterozygous mutations (arrow).

**Figure 4:**
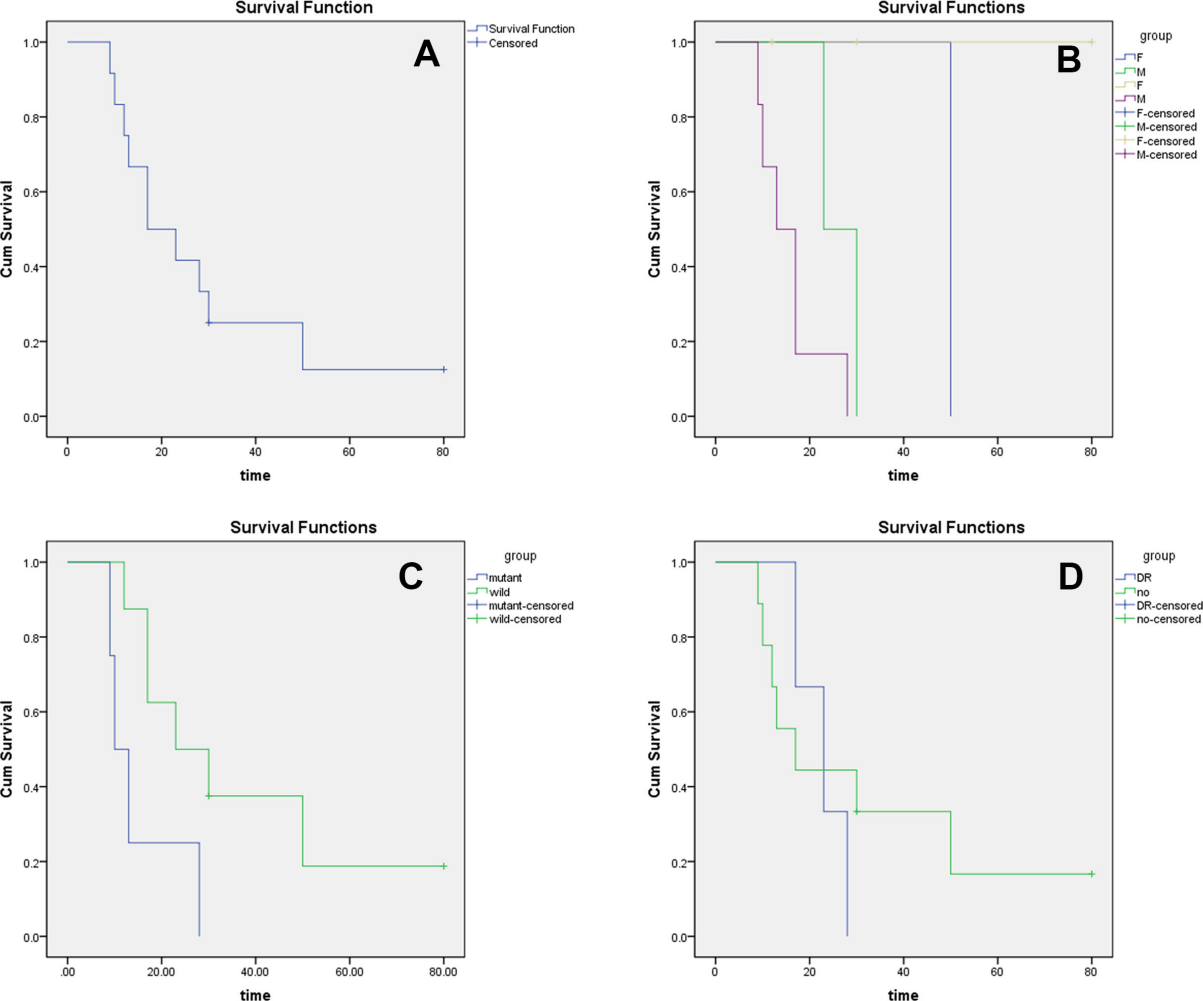
Survival analysis **(A)** It Showed that the 2-year overall survival rate was about 40%, and the 5-year overall survival rate was about 15%. **(B)** The survival rate of females (F) was significantly higher than that of males (M). **(C)** The prognosis of mutant MyoD1 was significantly worse than that of wild MyoD1. **(D)** Distant metastasis had no significant effect on survival.

Twelve adult patients with retroperitoneal spindle cell or sclerosing rhabdomyosarcoma were treated at Peking University International Hospital between 2014 and 2023. The clinical and pathological characteristics are summarized in **Table 1** and illustrated in **Figures 1**–**4**.

**Table 1:** Clinical and pathological characteristics and follow-up data of retroperitoneal SSRMS.

Case	Age/gender	Area	Clinical history	Diameter(cm)	MyoD1	Treatment	Recurrence	Follow-up
1	52/F	retroperitoneum	Retroperitoneal tumor recurred 6 months after surgery.	25	wild	surgery	LR	12m, DOD
2	42/M	retroperitoneum	The right back pain lasted for more than 2 months, and the physical examination found a retroperitoneal mass 2 days.	18	Mutant	surgery	LR	10m, DOD
3	51/F	retroperitoneum	Retroperitoneal tumor recured more than 1 month after surgery 4 months.	14	wild	surgery	LR3次	50m, DOD
4	35/M	Retroperitoneum, pelvic cavity	Retroperitoneal tumor had a recurrence 10 days after surgery 5 months.	19	Mutant	surgery	LR	9m, DOD
5	38/M	Retroperitoneum, pelvic cavity	The right low back pain lasted for more than 5 months. Retroperitoneal and pelvic tumor had a recurrence 2 days after surgery 4 months.	17	wild	surgery	LR, DR	17m, DOD
6	57/M	Retroperitoneum	The left lower limb was painful 3 months, the left retroperitoneal tumor was found more than 1 month.	30	Mutant	surgery	LR	13m, DOD
7	56/F	Retroperitoneum, mesenterium	Retroperitoneal tumor had a recurrence 3 years after surgery 4 years.	16	wild	Surgery, chemotherapy and targeted therapy	LR	80m, Alive
8	60/M	Retroperitoneum	Right retroperitoneal tumor was found for 5 months.	30	wild	Surgery, chemotherapy, targeted therapy and immunotherapy	LR	17m, DOD
9	48/M	Retroperitoneum	Retroperitoneal tumor had a recurrence 15 days after surgery 8 months.	12	wild	Surgery, radiotherapy and targeted therapy	LR、DR	23m, DOD
10	22/M	Retroperitoneum	He was admitted to the hospital after stopping defecation about half a month.	18	wild	Surgery	LR	30m, DOD
11	24/M	Retroperitoneum	The lower back was painful 10 months; he was diagnosed rhabdomyosarcoma about 9 months.	27	Mutant	Surgery, chemotherapy and interventional therapy	LR, DR	28m, DOD
12	58/F	Retroperitoneum	Retroperitoneal rhabdomyosarcoma had a recurrence 1 month after surgery 2 years.	38	wild	Surgery	LR	30m, Alive

Local Recurrence, (LR); Distant Recurrence and metastasis, (DR); Die of a Disease, (DOD).

#### 3.1.1 Clinical characteristics

There were eight male and four female patients (male-to-female ratio 2:1), with a median age of 51 years (range, 22–60) and a mean age of 44 years. All patients underwent surgical resection. Notably, extensive multiorgan involvement necessitated combined resections in multiple cases: partial resections of pancreas (n=1), ureter (n=2), large intestine (n=4), bladder (n=2), prostate (n=1), kidney (n=1), and small intestine (n=2). Six patients underwent primary surgery at our institution, while five underwent surgery for recurrent disease and one for a third recurrence.

Eight patients received surgery alone, while four received multimodal therapy that included chemotherapy, radiotherapy, targeted therapy, immunotherapy, or interventional therapy either preoperatively or postoperatively. At the time of first operation, no patient had nodal or distant metastases. Ten tumors measured >15 cm and were staged as T4, while two tumors were staged as T3 (>10 cm but ≤15 cm). All tumors were classified as high-grade, and patients were assigned to clinical stage III.

#### 3.1.2 Pathological findings

Grossly, tumors were nodular or multinodular, with solid or cystic components and gray-white to yellow cut surfaces. Tumor diameters ranged from 12 to 38 cm (mean: 22 cm). Microscopically, all tumors showed fascicular spindle cell and/or sclerotic morphology. Areas of necrosis were frequent. Spindle cells exhibited eosinophilic cytoplasm, resembling myofibroblastic or smooth muscle neoplasms, often with identifiable striated myoblasts. Sclerotic areas demonstrated hyalinization and dense collagen deposition. Atypia was moderate to severe, and mitotic figures ranged from 2 to 25 per 10 high-power fields. Tumors exhibited infiltrative margins or invasion into adjacent organs in all cases.

#### 3.1.3 Immunohistochemical profile

All tumors stained positive for Desmin, MyoD1, and/or myogenin. MyoD1 expression was significantly more frequent than myogenin (11/12 vs. 5/12, P<0.05). SMA was focally or diffusely positive in 5 tumors. P16 and P53 were positive in 7 and 6 cases, respectively. In contrast, all tumors were negative for CD34, S-100, MDM2, CDK4, and H3K27Me3.

#### 3.1.4 MYOD1 mutation analysis

Sanger sequencing of MYOD1 exon 1 revealed mutations in 4 of 12 cases (33.3%). One case harbored the canonical L122R hotspot mutation (c.365T>G), while the remaining three had rare variants: one nonsynonymous mutation (p.D47Y) and two synonymous substitutions (p.D78= and p.H70=). All mutations were somatic in origin.

#### 3.1.5 Follow-up and prognosis

All 12 patients experienced local recurrence during follow-up, and 3 patients (25%) developed distant metastases to the lung, liver, or spine. The median follow-up was 26.6 months (range, 9–80). Ten patients (83.3%) died of disease. Median overall survival was 17 months (range, 9–50). Two female patients were alive at the time of last follow-up (30 and 80 months, respectively).

#### 3.1.6 Survival analysis

Kaplan–Meier analysis showed a 2-year survival rate of 40% and a 5-year survival rate of 15% (**Figure 4A**). Female patients demonstrated significantly better survival compared to males (P<0.05; **Figure 4B**). MYOD1 mutation status was also significantly associated with survival (P<0.05; **Figure 4C**). The presence of distant metastasis, however, was not significantly associated with reduced survival (P>0.05; **Figure 4D**).

### 3.2 Individual patient data meta-analysis

**Figure 5:**
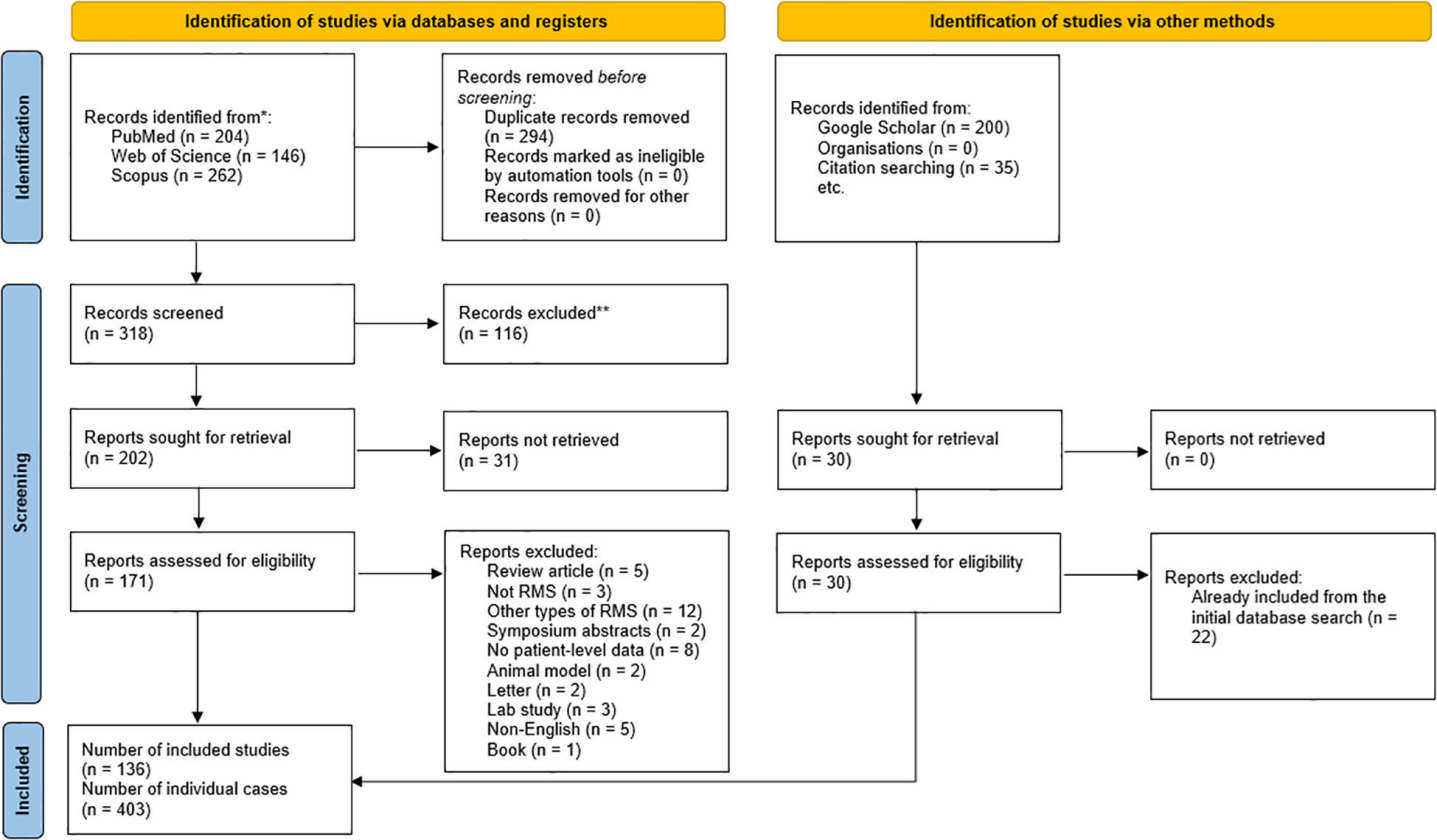
A PRISMA flow diagram showing the results of the database search.

A total of 136 studies—comprising 107 case reports and 29 case series—were included in the IPD meta-analysis, yielding 403 unique cases of ScRMS/SRMS. The PRISMA flowchart outlining the study selection process is presented in **Figure 5**.A full list of included studies can be found in **Supplementary Appendix**.

#### 3.2.1 Geographic and temporal trends

**Figure 6:**
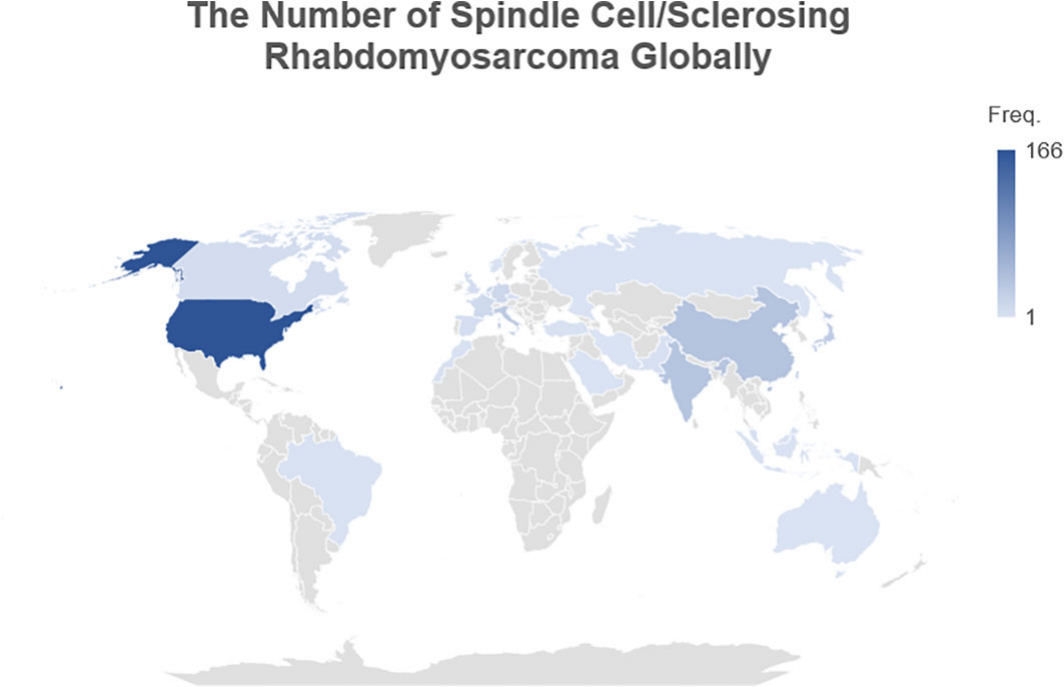
A global heatmap showing the frequency of spindle cell/sclerosing rhabdomyosarcoma cases worldwide.

Twenty-nine countries reported cases of ScRMS/SRMS. A global heatmap shows the frequency of reported cases globally (**Figure 6**). The United States accounting for the highest rate (166, 41.19%), followed by China (37, 9.18%), India (35, 8.68%), Italy (28, 6.95%), and Japan (25, 6.20%), respectively. Meanwhile, single cases were observed in Australia, Indonesia, Russia, Singapore, Norway, Slovenia, and Saudi Arabia. There were no reports from most of African and Middle Eastern countries.

**Figure 7:**
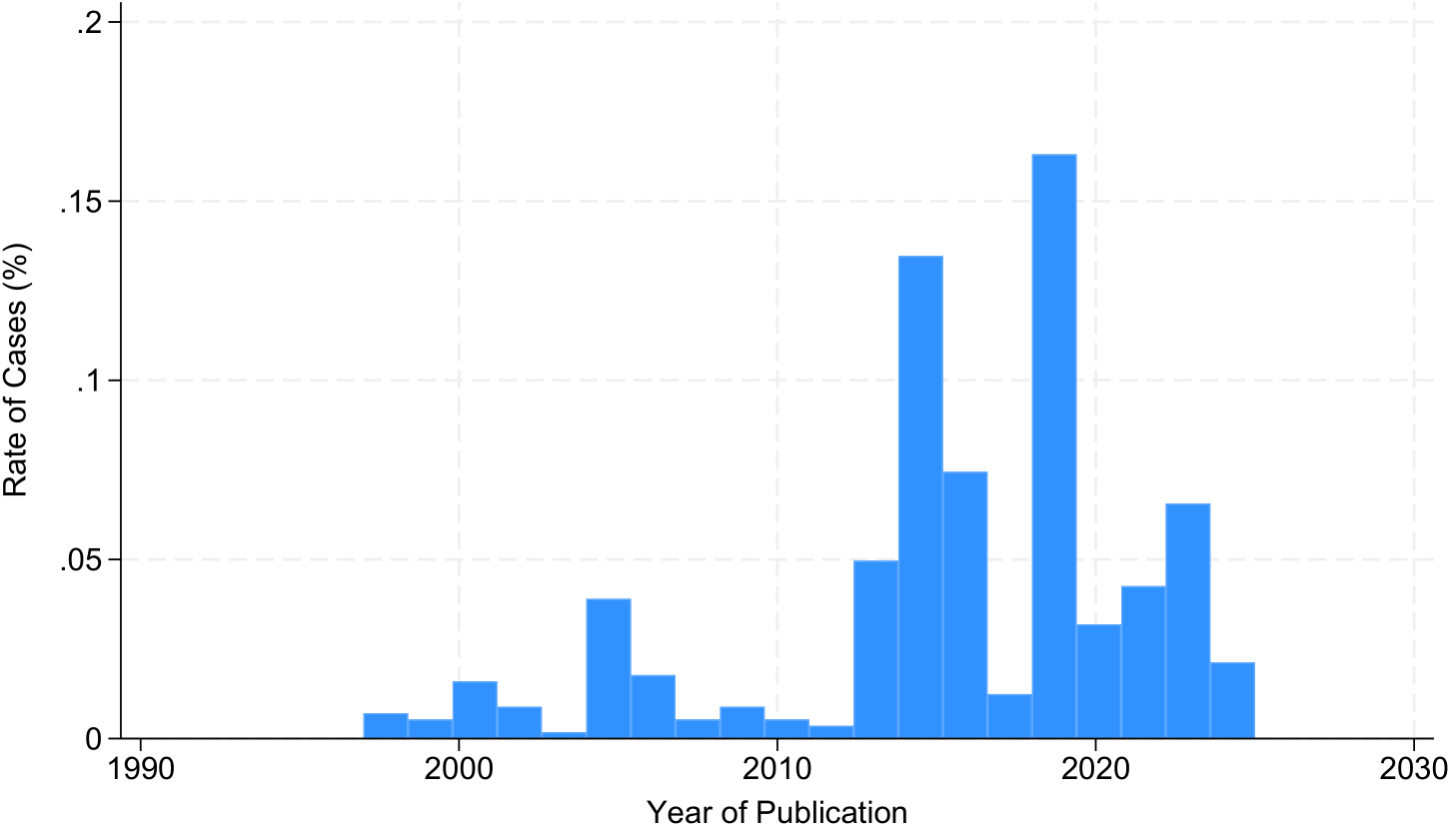
Temporal trends of the reporting rate of spindle cell/sclerosing rhabdomyosarcoma cases globally.

Time-wise analysis showed a progressive rise in the number of cases over the time from 2000 to 2025, with a peak rate in 2019 (68 cases, 16.87%). In 2022, 20 cases were reported (4.96%), and in 2023, 37 cases were reported (9.18%) (**Figure 7**).

#### 3.2.2 Demographic and clinical characteristics

**Figure 8:**
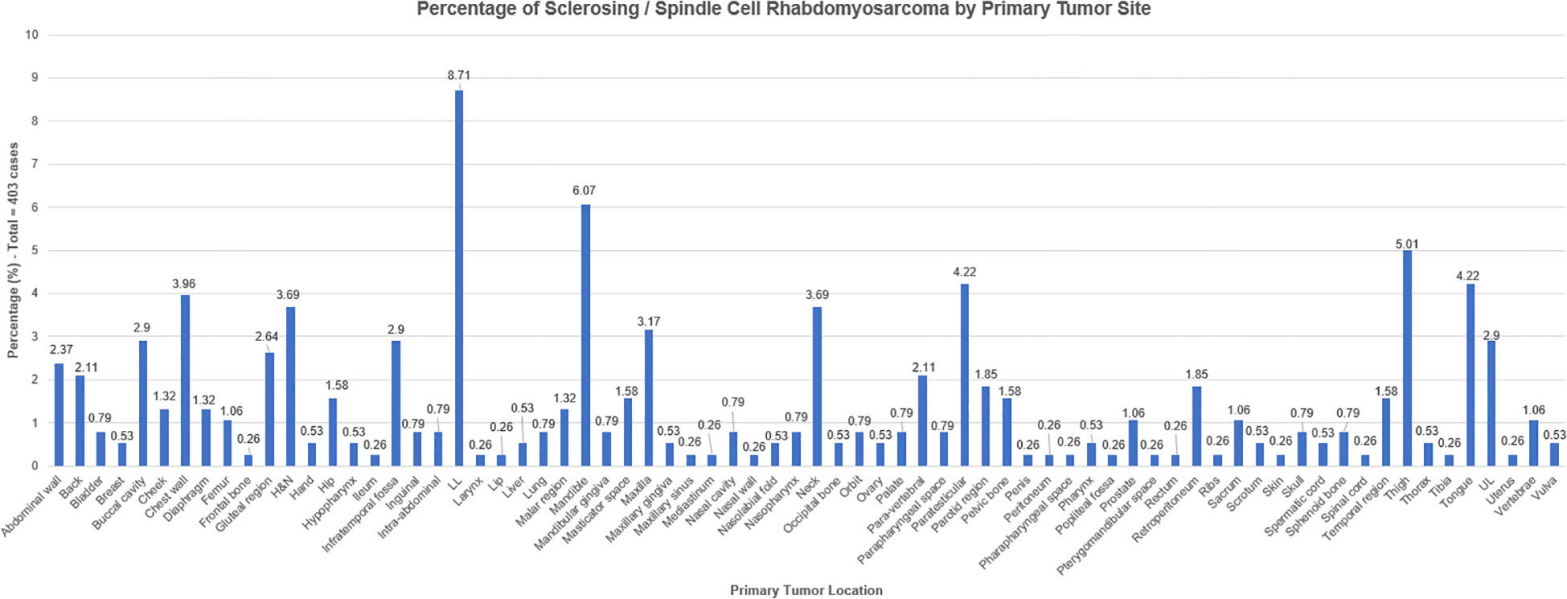
Tumor location-wise presentation of the frequency of spindle cell/sclerosing rhabdomyosarcoma cases globally.

Patients’ clinicodemographic data is presented in **Table 1**. The mean age of patients was 28.5 years (SD, 21.3), with a median of 25 years (IQR, 12–41). Of the 401 patients with available gender data, 55.9% were male (n, 224) and 44.1% were female (n, 177). Laterality was reported in 111 cases, with a slight predominance on the left (58.9%). The final diagnosis was sclerosing RMS in 84 cases (20.84%), spindle cell RMS in 266 cases (66%), and spindle cell/sclerosing RMS in 53 cases (13.15%). In terms of location, 72 tumor sites were reported, with the lower limb accounting for the most frequent site (33, 8.71%), followed by the mandible (23, 6.07%), paratesticular region (16, 4.22%), and the tongue (16, 4.22%), respectively (**Figure 8**).

Tumor size was available for 198 patients, with a mean diameter of 7.8 cm (SD, 6.9) and a median of 5.5 cm (IQR, 3.5–10.0). Mitoses per 10 high-power fields (HPFs) were reported in 60 patients, with a median of 9.5 (IQR, 4–18.5). The most common primary treatment modality was surgery (64.5%), followed by chemotherapy (19.2%) and chemoradiation (14.5%). Median follow-up duration was 14 months (IQR, 8–28). The mean Ki67 index (per 1000 cells) was reported in 37 cases, with a mean value of 26.35 (SD 21.75) and a median of 20 [IQR 10-35].

#### 3.2.3 Radiological findings

Magnetic resonance imaging (MRI) findings were inconsistently reported. Among 19 cases with T2-weighted (T2W) imaging, the majority (78.9%) showed hyperintensity (**Table 2**). T1-weighted (T1W) data were available for 18 cases, of which 66.7% were isointense. Contrast-enhanced CT findings (n, 40) were heterogeneous in 27.3% and hypervascular in 2.5%, with contrast enhancement present in over half of the cases (57.5%).

**Table 2:** Baseline clinicodemographic characteristics of patients with sclerosing/spindle cell rhabdomyosarcoma included in the individual patient-data meta-analysis.

Characteristic	Frequency/mean	Percent/SD
Age	n, 401	
Mean (SD)	28.5	21.3
Median [IQR]	25	[12 - 41]
Gender	n, 401	
Female	177	44.14
Male	224	55.86
Laterality	n, 111	
Left	66	58.93
Right	45	40.18
Tumor Size (cm)	n, 198	
Mean (SD)	7.77	6.91
Median [IQR]	5.5	[3.5 - 10]
Mitoses/10HPFs	n, 60	
Mean (SD)	13.44	11.93
Median [IQR]	9.5	[4 - 18.5]
T2W Imaging	n, 19	
Hyperdense	15	78.95
Heterogeneous	1	5.26
Isodense	2	10.53
Hypodense	1	5.26
T1W Imaging	n, 18	
Different enhancement	1	5.56
Heterogeneous	1	5.56
Hyperdense	1	5.56
Isodense	12	66.68
Hypodense	3	16.67
CT Contrast Enhancement	n, 40	
Yes	23	57.5
No	2	5
Hypodense	1	2.5
Homogenous	1	2.5
Hypervascular	1	2.5
Weak vasculature	1	2.5
Heterogeneous	11	27.25
Ki67	n, 37	
Mean (SD)	26.35	21.75
Median [IQR]	20	[10 - 35]
Primary Therapy	n, 276	
CRT	40	14.49
CT	53	19.2
RT	5	1.81
Surgery	178	64.49
Follow-up (months)	n, 267	
Mean (SD)	24.67	31.17
Median [IQR}	14	[8 - 28]
Clinical Outcome	n, 275	
AWD	79	28.21
AWOD	14	5
DOD	97	34.65
NED	85	30.36
Recurrence	n, 192	
No	112	58.33
Yes	80	41.67
Metastasis	n, 177	
No	123	69.49
Yes	54	30.51

SD, standard deviation; IQR, interquartile range; CRT, chemoradiation therapy; CT, chemotherapy; RT, radiotherapy; AWD, alive with disease; AWOD, alive without disease; DOD, died of disease; NED, no evidence of disease.

#### 3.2.4 Immunohistochemical profile

A detailed immunohistochemical (IHC) profile is shown in **Table 3**. Desmin (96.8% out 246 cases), MyoD1 (91% out of 200 cases), Myogenin (83.6% out of 189 cases), and MYF4 (96.2% out of 26 cases) were the most frequently positive muscle markers. S100 was variably expressed (21.6% out of 116 cases), while pancytokeratin (32 cases) and SMA (117 cases) showed positivity in 62.5% each. Several epithelial, neural, and melanocytic markers were tested but rarely positive. WT1 expression was consistently observed in all 11 cases tested. Meanwhile, these markers showed a low positivity rate: H-caldesmon (8.7% out of 23 cases), CD31 (5.56% out of 18 cases), CD34 (8.82% out of 68 cases), and EMA (9.76% out of 41 cases). CD99 (39.5% out of 38 cases) and CK AE1/AE3 (46% out of 50 cases) were found positive in approximately half of the tested population.

**Table 3:** Immunohistochemical profiles of sclerosing and spindle cell rhabdomyosarcoma cases included in the IPD meta-analysis.

IHC results	Positive result	Total observations	Percent (%)
Desmin	238	246	96.75
Myogenin	158	189	83.6
Nestin	1	1	100
Vimentin	30	30	100
H-caldesmon	2	23	8.7
HHF-35 (muscle-specific actin)	38	40	95
Fast myosin	5	6	83.33
Myoglobin	12	16	75
MyoD1	182	200	91
Mdm2	4	5	80
HBA 71	1	1	100
ALK-1	35	44	79.55
MYF 4	25	26	96.15
CD10	1	2	50
CD30	0	9	0
CD31	1	18	5.56
CD34	6	68	8.82
CD45	0	5	0
CD56	3	3	100
CD68	0	5	0
CD99	15	38	39.47
CD117	1	5	20
CD163	0	2	0
BCL2	1	1	100
D2-40	1	1	100
SOX10	1	24	4.17
S100 protein	25	116	21.55
Pancytokeratin	20	32	62.5
Calponin	1	3	33.33
SMA	70	112	62.5
EMA	4	41	9.76
Cytokeratin	3	25	12
NSE	0	3	0
CK AE1/AE3	23	50	46
MNF-116	0	1	0
CK18	1	9	11.11
CK20	0	4	0
CAM5.2	15	25	60
CK5/6	0	12	0
CK7	3	6	50
Fli-1	0	1	0
CK8	1	5	20
P40	0	3	0
ERG	0	7	0
Melan-A	0	5	0
HMB45	0	12	0
SALL4	0	1	0
Inhibin	0	1	0
GFAP	0	10	0
WT1	11	11	100
P53	2	3	66.67
P63	0	17	0
STAT6	0	3	0
Chromogranin	0	1	0
TLE1	0	4	0
TFE3	0	2	0
MUC4	0	2	0

IHC , immunohistochemistry; ALK-1 , anaplastic lymphoma kinase; BCL2 , B-cell lymphoma 2; CAM5.2 , cytokeratin antibody clone 5.2; CD10 , common acute lymphoblastic leukemia antigen; CD30 , cluster of differentiation 30; CD31 , platelet endothelial cell adhesion molecule; CD34 , cluster of differentiation 34; CD45 , leukocyte common antigen; CD56 , neural cell adhesion molecule; CD68 , cluster of differentiation 68; CD99 , MIC2 gene product; CD117 , c-Kit (proto-oncogene receptor tyrosine kinase); CD163 , cluster of differentiation 163 (macrophage marker); CK , cytokeratin; CK5/6 , cytokeratin 5 and 6; CK7 , cytokeratin 7; CK8 , cytokeratin 8; CK18 , cytokeratin 18; CK20 , cytokeratin 20; D2-40 , podoplanin; EMA , epithelial membrane antigen; ERG , ETS-related gene; Fli-1 , Friend leukemia integration 1 transcription factor; GFAP , glial fibrillary acidic protein; HBA 71 , antibody clone targeting CD99; HMB45 , human melanoma black 45; IHC , immunohistochemistry; Mdm2 , murine double minute 2; MNF-116 , monoclonal antibody cocktail (cytokeratins); MUC4 , mucin 4; MYF4 , myogenic factor 4; NSE , neuron-specific enolase; P40 , ΔNp63 isoform; P53 , tumor protein p53; P63 , tumor protein p63; S100 , S100 calcium-binding protein; SALL4 , sal-like protein 4; SMA , smooth muscle actin; SOX10 , SRY-box transcription factor 10; STAT6 , signal transducer and activator of transcription 6; TFE3 , transcription factor E3; TLE1 , transducin-like enhancer of split 1; WT1 , Wilms tumor 1.

#### 3.2.5 Molecular findings

Of 111 cases tested for MYOD1, 95.5% harbored mutations, with homozygous L122R mutations identified in 26.4% and heterozygous mutations in 13.2% (**Table 4**). Wild-type MYOD1 was present in 21.7% of cases. PIK3CA mutations were universally detected among 77 tested cases (100% positivity rate). Other rare alterations included mutations in FGFR4, NRAS, ARID1A, and DICER1.

**Table 4:** Distribution of fusion types among fusion-positive SS/RMS cases in the IPD meta-analysis.

Fusion type	Number of positive cases	Positivity rate
DCTN1::ALK	1	1.43
EP300::VGLL3	2	2.86
ESWR1::TFCP2	13	18.57
EWSR1::UBP1	1	1.43
FUS::TFCP2	23	32.86
FUS::TFCP3	1	1.43
FUS::TFCP4	1	1.43
FUS-TFCP5	1	1.43
MEIS1::FOXO1	1	1.43
MEIS1::NCOA2	4	5.71
PAX8::PPARG	1	1.43
PLOD2::RBM6	1	1.43
SRF::NCOA2	1	1.43
TCF12::VGLL3	1	1.43
TEAD1::NCOA2	9	12.85
TFG::MET	1	1.43
VAX2::ALK	1	1.43
VGLL2::CITED2	4	5.71
VGLL2::NCOA2	2	2.86
YAP1::MAML2	1	1.43

ALK, Anaplastic Lymphoma Kinase; CITED2, CBP/P300-Interacting Transactivator With Glu/Asp-Rich Carboxy-Terminal Domain 2; DCTN1, Dynactin Subunit 1; EP300, E1A Binding Protein P300; EWSR1, Ewing Sarcoma Breakpoint Region 1; FOXO1, Forkhead Box O1; FUS, Fused in Sarcoma; MAML2, Mastermind-Like Transcriptional Coactivator 2; MEIS1, Meis Homeobox 1; MET, MET Proto-Oncogene, Receptor Tyrosine Kinase; NCOA2, Nuclear Receptor Coactivator 2; PAX8, Paired Box Gene 8; PLOD2, Procollagen-Lysine,2-Oxoglutarate 5-Dioxygenase 2; PPARG, Peroxisome Proliferator-Activated Receptor Gamma; RBM6, RNA Binding Motif Protein 6; SRF, Serum Response Factor; TCF12, Transcription Factor 12; TEAD1, TEA Domain Transcription Factor 1; TFCP2, Transcription Factor CP2; TFG, TRK-Fused Gene; UBP1, Upstream Binding Protein 1; VAX2, Ventral Anterior Homeobox 2; VGLL2, Vestigial Like Family Member 2; VGLL3, Vestigial Like Family Member 3; YAP1, Yes-Associated Protein 1.

Fusion gene data were available for a subset of patients (**Table 5**). The most prevalent fusion was FUS::TFCP2 (32.9%), followed by EWSR1::TFCP2 (18.6%) and TEAD1::NCOA2 (12.9%). Less common fusions included MEIS1::NCOA2, SRF::NCOA2, VGLL2::CITED2, and EP300::VGLL3.

**Table 5:** Fluorescence *in situ* hybridization (FISH) results for gene rearrangements and amplifications in SS/RMS cases in the IPD meta-analysis.

Gene/Region	Frequency	Percent (%)
ESWR1 rearrangement	n, 6	
Negative	1	16.67
Positive	5	83.33
FUS rearrangement	n, 5	
Negative	1	20
Positive	4	80
NCOA2 rearrangement	n, 22	
Negative	19	86.36
Positive	3	13.64
VGLL2 rearrangement	n, 2	
Negative	1	50
Positive	1	50
SRF rearrangement	n, 3	
Negative	2	66.67
Positive	1	33.33
PAX3 rearrangement	n, 18	
Negative	18	18
Positive	0	18
FOXO1 rearrangement	n, 16	
Negative	16	16
Positive	0	16
TEAD1 rearrangement	n, 1	
Negative	0	1
Positive	1	1
NCOA1 rearrangement	n, 18	
Negative	18	18
Positive	0	18
SYT gene amplification	n, 1	
Negative	1	1
Positive	0	1
MDM2 amplification	n, 5	
Negative	3	5
Positive	2	5
HGMA2 rearrangement	n, 1	
Negative	0	1
Positive	1	1
NYMC amplification	n, 1	
Negative	0	1
Positive	1	1
MYCN amplification	n, 1	
Negative	0	1
Positive	1	1

Positive, FISH confirmed gene rearrangement or amplification; Negative, No rearrangement or amplification detected; EWSR1, Ewing Sarcoma Breakpoint Region 1; FUS, Fused in Sarcoma; NCOA1/2, Nuclear Receptor Coactivator 1/2; VGLL2, Vestigial Like Family Member 2; SRF, Serum Response Factor; PAX3, Paired Box Gene 3; FOXO1, Forkhead Box O1; TEAD1, TEA Domain Transcription Factor 1; SYT, Synovial Sarcoma Translocation (SS18); MDM2, Murine Double Minute 2; HMGA2, High Mobility Group AT-Hook 2; NYMC, Likely refers to New York Medical College–linked probe (clarify if this is a placeholder); MYCN, MYCN Proto-Oncogene, BHLH Transcription Factor.

FISH analysis confirmed gene rearrangements in several key fusions (**Table 6**), including FUS, EWSR1, NCOA2, SRF, and VGLL2. Rearrangements in PAX3, FOXO1, and NCOA1 were uniformly negative across tested samples, supporting their rarity in this RMS subtype.

**Table 6:** Genomic alterations identified in SS/RMS cases: frequency and positivity rate by mutation type in the IPD meta-analysis.

Mutation/alteration type	Positive frequency	Total cases	Positivity rate (%)
MYOD1	106	111	95.5
L122R heterozygous	14	106	13.21
L122R homozygous	28	106	26.42
Wild type	23	106	21.7
Not classified	41	106	38.68
FGFR4	2	25	8
PIK3CA	77	77	100
NRAS	1	1	100
ARID1A	1	1	100
PTCH1	1	1	100
FANCC	1	1	100
Triosomy 13	1	1	100
CDKN21/CDKN2B deletion	1	1	100
DOT1L	1	1	100
DICER1	1	1	100

MYOD1, Myogenic Differentiation 1; FGFR4, Fibroblast Growth Factor Receptor 4; PIK3CA, Phosphatidylinositol-4,5-Bisphosphate 3-Kinase Catalytic Subunit Alpha; NRAS, Neuroblastoma RAS Viral Oncogene Homolog; ARID1A, AT-Rich Interaction Domain 1A; PTCH1, Patched 1; FANCC, Fanconi Anemia Complementation Group C; Trisomy 13, Gain of chromosome 13; CDKN2A/CDKN2B, Cyclin-Dependent Kinase Inhibitor 2A/2B; DOT1L, Disruptor of Telomeric Silencing 1-Like; DICER1, Dicer 1, Ribonuclease III; L122R, Point mutation at codon 122 in MYOD1 resulting in leucine-to-arginine substitution.

#### 3.2.6 Clinical outcome

Of the 275 patients with available outcome data, 34.7% died of disease (DOD), while 30.4% had no evidence of disease (NED) at last follow-up. Alive with disease (AWD) was documented in 28.2%, and only 5% were alive without disease (AWOD). Local recurrence occurred in 41.7% (80 of 192), and metastasis was documented in 30.5% (54 of 177).

## 4 Discussion

Spindle cell and sclerosing rhabdomyosarcoma (Sc/SRMS) remains one of the rarest and most histologically deceptive soft tissue sarcomas, with its diagnosis, treatment, and prognosis presenting unique challenges (5, 28–34). In this study, we report one of the largest institutional case series to date of retroperitoneal Sc/SRMS and combine these data with an IPD meta-analysis of 403 published cases to provide the most comprehensive characterization of this rare entity. Our findings yield several insights with direct clinical and translational relevance.

Our institutional series of 12 retroperitoneal Sc/SRMS cases reinforces the aggressive clinical behavior of this subtype, with a striking 100% rate of local recurrence and a 2-year overall survival (OS) of only 40%. These outcomes compare unfavorably even against the broader IPD meta-analysis population, in which approximately one-third of patients died of disease and recurrence was observed in 41.7%. Retroperitoneal tumors in particular are prone to extensive local invasion, often requiring multiorgan resections, as reflected in our cohort (22–24). This underscores the need for more aggressive local control strategies and long-term surveillance for these anatomically deep-seated lesions.

In our institutional cohort, all 12 patients presented with retroperitoneal Sc/SRMS, allowing for a direct comparison with the six retroperitoneal cases identified in the IPD meta-analysis (21–26). Notably, the median age among IPD retroperitoneal cases was considerably younger (37 years, range: 0.33–53) compared to our institutional series (median: 51 years), suggesting potential biological or reporting differences. Both datasets demonstrated a male predominance (IPD: 4 males, 2 females; institutional: 8 males, 4 females), and tumor sizes in both cohorts were consistently large, often exceeding 10 cm. While all institutional cases underwent surgery and had molecular testing, none of the IPD retroperitoneal cases reported fusion or genetic profiling—highlighting a critical gap in historical reporting. Recurrence data were variably available in the IPD set, but our institutional series demonstrated a uniform pattern of local recurrence and a markedly high mortality rate (83.3%), reinforcing the aggressive nature of retroperitoneal tumors (10, 35–37). The absence of detailed molecular data in published cases underscores the value of our cohort in contributing to the molecular characterization of this underreported anatomical subset.

Histologically, all cases in our series displayed the hallmark spindle and/or sclerotic architecture, with moderate to severe atypia and frequent mitoses—features consistent with prior reports (38–41). Immunophenotypically, universal expression of Desmin and MyoD1, and to a lesser extent myogenin, confirms the myogenic differentiation of these tumors. Notably, MyoD1 was significantly more prevalent than myogenin (92% vs. 42%), echoing findings in the pooled IPD analysis and supporting the former’s greater diagnostic sensitivity in Sc/SRMS (42). Loss of H3K27Me3 (43), commonly associated with malignant peripheral nerve sheath tumors, was absent across all institutional cases, aiding in the exclusion of differential diagnoses.

A major highlight of our study lies in the integration of molecular findings. In our cohort, one-third of patients harbored MYOD1 mutations, including both the canonical L122R variant and rare mutations. Within the IPD meta-analysis, MYOD1 mutations were present in over 95% of tested cases, affirming its pivotal role in Sc/SRMS oncogenesis (12, 13, 44). The pathogenic relevance of MYOD1 is further underscored by its strong association with adverse outcomes in our survival analysis, in line with prior studies suggesting MYOD1-mutant RMS constitutes a distinct, high-risk subgroup. Furthermore, our analysis revealed a universal co-occurrence of PIK3CA mutations among MYOD1-mutant cases (45), suggesting a potential cooperative oncogenic mechanism that may represent a therapeutic vulnerability.

Fusion gene analysis also uncovered consistent patterns, with FUS::TFCP2 and EWSR1::TFCP2 being the most frequent alterations, found in 33% and 19% of cases, respectively. These TFCP2 fusions appear specific to Sc/SRMS and were notably absent in other rhabdomyosarcoma subtypes. The frequency of such fusions in our IPD data supports their diagnostic utility and the need for routine screening, particularly in histologically ambiguous cases. Meanwhile, fusions involving PAX3, FOXO1, and NCOA1—canonical to alveolar RMS—were uniformly absent, further delineating the molecular boundary of Sc/SRMS (46–48).

Radiologically, despite limited and heterogeneous reporting, we found that hyperintensity on T2-weighted MRI was present in over 75% of reported cases, consistent with the high cellularity and myxoid stroma seen histologically. However, the limited utility of imaging in distinguishing Sc/SRMS from other spindle cell neoplasms reiterates the central role of tissue diagnosis and comprehensive immunohistochemical and molecular profiling (49).

Our findings also raise important considerations regarding sex-related survival differences. In our institutional series, female patients showed significantly better survival, and all long-term survivors were women. While the IPD meta-analysis did not demonstrate a statistically significant sex disparity, this observation warrants further investigation, potentially reflecting underlying biological differences or treatment responsiveness.

Despite the strength of combining institutional data with an IPD-level synthesis of the literature, this study is not without limitations. First, the retrospective nature of both our institutional cohort and the included literature introduces inherent biases, including variability in reporting, treatment regimens, and follow-up duration. Second, while our institutional series provides valuable clinical and pathological data on retroperitoneal Sc/SRMS, the molecular analysis was limited to MYOD1 mutation testing. Other recurrent genetic alterations, such as PIK3CA mutations and gene fusions, which were prominent in the IPD meta-analysis, were not assessed in our institutional cohort. This limits the depth of molecular comparison between the two datasets. Third, the molecular testing panels were inconsistent across studies, limiting the generalizability of mutation frequency estimates for less commonly reported genes. In addition, the observed survival advantage among female patients should be interpreted cautiously. Although this finding reached statistical significance in the institutional cohort, it was not reproduced in the pooled meta-analysis and may reflect the limited sample size, particularly the small number of female patients (n, 12), rather than a true biological difference. In addition, although PIK3CA mutations were identified in 100% of tested cases in the IPD analysis, this proportion applies only to the subset of patients who underwent molecular testing (77 cases) and should not be interpreted as representing the entire study population. Finally, as this IPD analysis is based on case reports and small case series, it is subject to inherent publication bias and substantial reporting variability across studies. These factors precluded formal quantitative synthesis and limited inferential statistical comparisons across subgroups, restricting our analyses to descriptive presentation of available data and limiting the generalizability of the findings.

Nevertheless, this study offers a novel, data-driven foundation to inform future research and clinical management of Sc/SRMS. Routine MYOD1 sequencing and fusion testing should be considered essential for diagnosis, prognostication, and future therapeutic stratification. The high rate of local recurrence and mortality—especially among patients with retroperitoneal involvement—emphasizes the need for multimodal strategies that combine surgical aggressiveness with tailored systemic therapies. In particular, the consistent presence of PIK3CA mutations across MYOD1-mutant tumors suggests a potentially targetable axis that warrants further preclinical and clinical exploration. It is important to note, however, that the high frequencies of MYOD1 and PIK3CA mutations reported in the meta-analysis are based on a subset of tested cases; these findings should therefore be interpreted as representative of the molecularly characterized subgroup rather than the entire Sc/SRMS population, and more comprehensive and systematic molecular profiling in future prospective studies is needed.

## Data Availability

The original contributions presented in the study are included in the article/**Supplementary Material**. Further inquiries can be directed to the corresponding author.
